# Activation of MEK‐ERK‐c‐MYC signaling pathway promotes splenic M2-like macrophage polarization to inhibit PHcH-liver cirrhosis

**DOI:** 10.3389/fimmu.2024.1417521

**Published:** 2024-11-08

**Authors:** Wang Guihu, Dong Wei, Zhang Hailong, Zhang Chongyu, Sun Jin, Zhu Mengchen, Zhang Jian, Zhou Rui, Ren Song, Zhang Chen, Liu Xi, Li Zongfang, Jiang An

**Affiliations:** ^1^ National and Local Joint Engineering Research Center of Biodiagnostics and Biotherapy, Second Affiliated Hospital, Xi’an Jiaotong University, Xi’an, China; ^2^ Shaanxi Provincial Clinical Medical Research Center for Liver and Spleen Diseases, The Second Affiliated Hospital, Xi’an Jiaotong University, Xi’an, China; ^3^ Department of Hepatobiliary pancreas surgery and liver transplantation, The Second Affiliated Hospital, Xi’an Jiaotong University, Xi’an, China

**Keywords:** macrophages, c-Myc, MAPK, portal hypertension, hypersplenism, liver cirrhosis

## Abstract

**Introduction:**

Portal hypertension combined with hypersplenism (PHcH) is the main cause of hypocytosis and esophagogastric variceal hemorrhage in patients with liver cirrhosis. Activated macrophages that destroy excess blood cells are the main cause of hypersplenism, but the activating pathway is not very clear. This study aims to investigate the activation types of splenic macrophages and their activation mechanisms, to provide experimental evidence for the biological treatment of splenomegaly, and to find a strategy to improve liver fibrosis and inflammation by intervening in splenic immune cells. This study revealed the occurrence of M2-like polarization of macrophages and upregulation of c-Myc gene expression in the PH spleen.

**Methods:**

RNAseq, protein chip, western blot, and chip-seq were performed on macrophages and the *in vitro* MEK inhibitor rafametinib was used. Carbon tetrachloride and thioacetamide induced mouse cirrhosis models were separately constructed.

**Results:**

c-Myc gene knockout in splenic macrophages reduced M2-like polarization and exacerbated liver fibrosis inflammation. c-Myc activated the MAPK signaling pathway and upregulated the expression of IL-4 and M2-like related genes in PH hypersplenism through the MEK-ERK-c-Myc axis. In addition, the c-Myc gene exerted anti-inflammatory effects by upregulating IL-4-mediated signal transduction to promote M2-like differentiation and anti-inflammatory cytokine secretion.

**Conclusions:**

Activation of MEK‐ERK‐c‐MYC signaling pathway promotes splenic M2-like macrophage polarization to inhibit PHcH-liver cirrhosis. Therefore, the induction of macrophage depolarization might represent a new therapeutic approach in the cure of PH hypersplenism, making c-Myc a potential candidate for macrophage polarization therapy.

## 1 Introduction

Cirrhotic portal hypertension (PH) is a clinical syndrome characterized by portal collateral circulation, splenomegaly, and ascites (1). Patients often show hypersplenism, which is a common and frequently occurring complication in China. Although the pathogenesis of hypersplenism is still unclear, splenic macrophage phagocytosis, destruction, and hemocytosis are involved in its pathogenesis (2, 3). Macrophages are innate immune cells participating in homeostasis and defense. This depends on their activated phenotypes, including the typical macrophage phenotype (M1-like) and the alternatively activated macrophage (M2-like) (4, 5). Previous studies reported that activation of splenic macrophages is involved in liver cirrhosis-related splenic hyperfunction in PH (3, 6, 7).

Transcription factors are involved in the polarization of macrophages, which secrete IL-12 or IL-10, both regulatory cytokines for the production of IFNγ and development of Th1 cells, called M1 polarization (4, 8, 9). M2 macrophages are activated by helper T cytokines 2 (Th2) such as IL-4 and IL-13 to induce high expression of arginase-1 (arg1), while also producing higher levels of the anti-inflammatory cytokines such as mannose receptor-1 (Mrc1), and IL-10 involved in tissue repair (5, 10–13). The MYC protooncogene is represented downstream of receptor signal transduction pathways, resulting in positive or negative regulation of the MYC gene. c-Myc controls 45% of the genes related to M2 macrophage activation, including the direct upregulation of SCARB1, ALOX15, and MRC1 expression (14). IL-4 induces the transformation of M1 macrophages to the M2 phenotype by up regulating the expression of JNK and its downstream transcription factor c-myc (15). Our previous study on hypersplenism spleen transcription factor chip showed that the activity of c-Myc in hypersplenism spleen macrophages is significantly higher than that in normal spleen macrophages. The c-Myc gene is a transcription factor involved in the polarization of M2-like macrophages, but its role in the regulation of macrophage polarization in patients with PH hypersplenism and its mechanism have not yet been studied and reported.

Our team previously demonstrated the activation of the LPS/TLR/NF-κB signaling pathway in splenic macrophages with hypersplenism; their phagocytic function and antigen presentation function are stronger than normal cells (16). Mitogen activated protein kinases (MAPKs) transduce extracellular stimulation signals into cells and nuclei, leading to cell proliferation, differentiation, transformation, and apoptosis (17). Several studies have reported that MAPK pathway is involved in promoting M2-like macrophage activation and immune regulation (11, 18, 19). c-Myc is also involved in the activation of splenic macrophages in PH hypersplenism through the MAPK signaling pathway.

## 2 Materials and methods

### 2.1 Clinical spleen samples

Fresh human normal spleen samples (n = 15) were obtained from patients with traumatic splenic rupture who underwent splenectomy (Nor group); splenic samples (n = 36) of hepatitis B cirrhosis accompanied by PH were obtained from patients with PH hypersplenism who underwent splenectomy (PH group). The following individuals were excluded from the study: patients with hypersplenism caused by non-hepatitis B virus infection such as hepatitis C and autoimmune hepatitis and patients without hepatitis accompanied by tumor splenectomy. All procedures involving human sample collection were approved by the ethics committee of the Second Affiliated Hospital of Xi’an Jiaotong University (ID:2016127) and were performed according to the principles of the declaration of Helsinki. Signed informed consent was obtained by all individuals or their families.

### 2.2 Generation of genetically modified mice

The Cre gene in Lyz cre mice is mainly expressed in mature macrophages, and the Cre recombinase produced can mediate the cleavage of loxP on both sides of the c-Myc gene. c-Myc loxP**^+/+^** and Lyz cre^+/-^ mice used in this study were donated by the Department of pathology of the University of Pittsburgh in the United States. We selected Lyz cre^+/-^ to hybridize with c-Myc loxp^+/+^mice to obtain Lyz cre^+/-^ c-Myc loxp^+/-^, and then backcrossed with c-Myc loxp^+/+^mice to obtain mice with specific knockout of c-Myc in macrophages. The genotype was Lyz cre^+/-^ c-Myc loxp^+/+^(KO group), and the littermate c-Myc Flox^+/+^mice were used as controls (WT group).In this model, the fifth exon of c-Myc gene was knocked out. The primers used for genotype identification are shown in **Supplementary Table S3**.

### 2.3 Animals and treatment

All animal experimental protocols were approved by the Animal care and Use Committee of Xi’an Jiaotong University and carried out in accordance with the guidelines and recommendations issued by the National Institutes of health of China. C57BL/6 mice aged 6-8 weeks were purchased from the experimental animal center of Xi’an Jiaotong University (Xi’an, China). Two mouse liver fibrosis models were constructed using carbon tetrachloride (CCl4) and thioacetamide (TAA) for *in vivo* animal experiments: CCl4 induces mouse liver injury, and TAA simulates chronic liver fibrosis. CCl4 was intraperitoneally injected twice a week for 6 weeks (CCl4: olive oil = 1:3, 2 μL/g body weight), to establish the CCl4-induced liver fibrosis model (20). The hepatic fibrosis model induced by thioacetamide (TAA) solution was established by the administration of TAA (V900086; 300 mg/L; Sigma Aldrich), into the drinking water for 27 weeks (21).

### 2.4 Isolation of monocyte-macrophages by immunomagnetic beads

Density gradient centrifugation was used to obtain mononuclear cells from spleen samples. CD14 antigen is highly expressed in most monocyte-macrophages (22); thus patients’ spleen monocyte-macrophages were isolated using human CD14 MicroBeads (130-050-201; Milteny Biotec; Auburn, CA). Mouse spleen monocyte-macrophages were purified and isolated using CD11b MicroBeads (130-093-634; Milteny Biotec; Auburn, CA). The protocol steps for sorting monocyte-macrophages were provided by the instructions of immune magnetic beads of Milteny Biotec.

### 2.5 Hematoxylin-eosin and Sirius red staining

Hematoxylin-eosin staining was performed on the liver and spleen samples of mice in different groups of the two models to evaluate the pathological changes of liver and spleen. Sirius red staining was used to evaluate liver injury and collagen fiber deposition. At least five different regions of each mouse were selected for image acquisition and quantified by Image Pro Plus 6.0 software. At least 5-10 mice were included in the drug treatment group and KO group.

### 2.6 Immunofluorescence

The proportion of CD206^+^ M2-like macrophages in the spleen of patients was evaluated by double immunofluorescence using rabbit anti-CD11b antibody (1:200; ab133357; Abcam) and mouse anti-CD206 (1:100; sc-58986; SANTA CRUZ),. Evaluation of the proportion of CD206^+^ M2 like macrophages in mouse spleen using rabbit anti-F4/80 antibody (1:200; ab300421; Abcam) and mouse anti-CD206. Simultaneously setting up a blank control and a negative control without primary antibody. Followed by the second antibody CoraLite488-reconciled goat anti-rabbit IgG (1:100; SA00013-2; Proteintech) and CoraLite594-reconciled goat anti-mouse IgG (1:100; SA00013-3; Proteintech).

### 2.7 Flow cytometry

Flow cytometry (FACS) was used to analyze the macrophage ratio in the spleen cells of patients and mice. The antibodies are shown in **Supplementary Table S1**. Intracytoplasmic staining was performed using an Intraprep permeabilization reagent (A07803; Beckman Coulter). BD-FACS Canto II cytometer (BD Bioscienses) was used to measure the cell count, which was analyzed with FlowJo software (FlowJo 10, LLC, Ashland, OR).

### 2.8 Western blot analysis

Total proteins were isolated from spleen tissue or macrophages cell samples using radio immunoprecipitation assay buffer (RIPA) according to the manufacturer’s instructions (Beyotime, China). Protein concentration was measured using the bicinchoninic acid (BCA) assay. Optical density was detected by Bio Rad imaging system. All the antibodies used in this study are listed in the Supporting **Supplementary Table S2**.

### 2.9 Quantitative real-time PCR

Total RNA was extracted from cells using Trizol Regent, and the amplification products were quantified and analyzed by SYBR premix ex Taq II (RR820A; Takara) and ABI 7500 rapid instruments (ABI Life technologies). GAPDH was used as the housekeeping gene for the calculation of relative gene expression. The primers used in this study are listed in Supporting **Supplementary Table S3**.

### 2.10 Protein phosphorylation array

Phosphorylation of MAPK signaling proteins (p-CREB, p-ERK1/2, p-GSK3a, p-GSK3b, p-MEK, and p-MSK2) was detected using a human/mouse MAPK pathway phosphorylation array C1 (#AAH-MAPK-1-8; RayBiotech) according to the manufacturer’s protocol. The gray value of each image was detected by ImageJ software. The negative control value (NEG mean) was removed from the positive samples to obtain the actual expression, and the ratio (phosphorylated protein/positive control group) was expressed as the relative protein expression of the positive control group.

### 2.11 Primary cell culture and MEK1/2 inhibitor

Fresh spleen samples were collected from patients with traumatic spleen and PH spleen, and spleen mononuclear cells were extracted. The number of mononuclear cells used for *in vitro* culture of each sample should not be less than 3×10^8^. The same sample was divided into a control group and MEK inhibitor group (HY-14691; Refametinib), and titrate the inhibitor concentration with CCK8 (abs50003; Absin), with an inhibitor dose of 47 nM. Both groups of cells were cultured at 37 °C for 18 hours. A small amount of cultured mononuclear cells was used for nucleic acid and protein validation. Most of the remaining cells were used to sort macrophages by CD11b magnetic beads and used for subsequent experiments.

### 2.12 RNA sequencing and date processing

RNA sequencing analysis was performed on 12 samples of splenic monocytesc-macrophages: 6 samples from traumatic spleen and 6 samples from PH spleen. The construction and sequencing of the cDNA library for all RNA samples were performed by Baimike Biotechnology Co., Ltd. Functional annotation and Kyoto Encyclopedia of genes and genomes (KEGG) analysis were performed on two groups of differentially expressed genes (DEGs). Two criteria were used to screen DEGs: (1) fold change greater than 1.5 and (2) corresponding adjusted *p* value less than 0.05.

### 2.13 Chromatin immunoprecipitation

Chromatin immunoprecipitation (Chip) assay was performed using One-Day Chromatin IP Kits (17-10086; EZ-Magna Chip™ A/G; Millipore),. The simple process is as follows: the monocyte-macrophages sorted by magnetic beads were treated with formaldehyde to crosslink the protein with DNA, and the chromatin was cut to the size of 200-1000 BP by an ultrasonic cell breaker. Rabbit anti-c-Myc antibody (#13987; CST), and rabbit derived negative control IgG (#2729; CST), were used. After reverse cross-linking, DNA was purified by purification column, using 5% sample as input. The purified DNA was controlled by ordinary PCR, and the expected size was 166 bp. In addition, the reported downstream genes HK2, NCL (23), PER1, CRY1 (24) and NPM1 (#4779; CST), were used for ChIP quality control by qRT-PCR. The primers are listed in **Supplementary Table S3**.

### 2.14 Chip sequencing

The c-Myc gene chip pull-down samples and input samples were sequenced, as well as one sample of normal trauma spleen and one sample of hypersplenism spleen by Kang Cheng Biotechnology Co., Ltd. The differentially enriched regions for Peak Promoter Annotation in the experimental group *versus* control group were analyzed, and the expression changes of c-Myc dependent genes in M2 macrophages were compared (14, 25); qPCR was performed on spleen macrophage samples to verify some regulatory genes involved in M2-like macrophage cell activation (14). The sequencing primers are listed in **Supplementary Table S3**.

### 2.15 Statistical analysis

All plotting and statistical analysis were performed using GraphPad prism 8 (GraphPad Software Inc., San Diego, CA). Normally distributed data were compared using Student’s *t*-test or one-way or two-way analysis of variance (ANOVA). Parametric statistical analysis of non-normally distributed data was performed using Mann Whitney U test. Results were expressed as mean ± standard deviation, and a value of *P* < 0.05 was considered statistically significant.

## 3 Results

### 3.1 c-Myc increased and M2-like macrophages were activated in the spleen of PH patients

**Figure 1:**
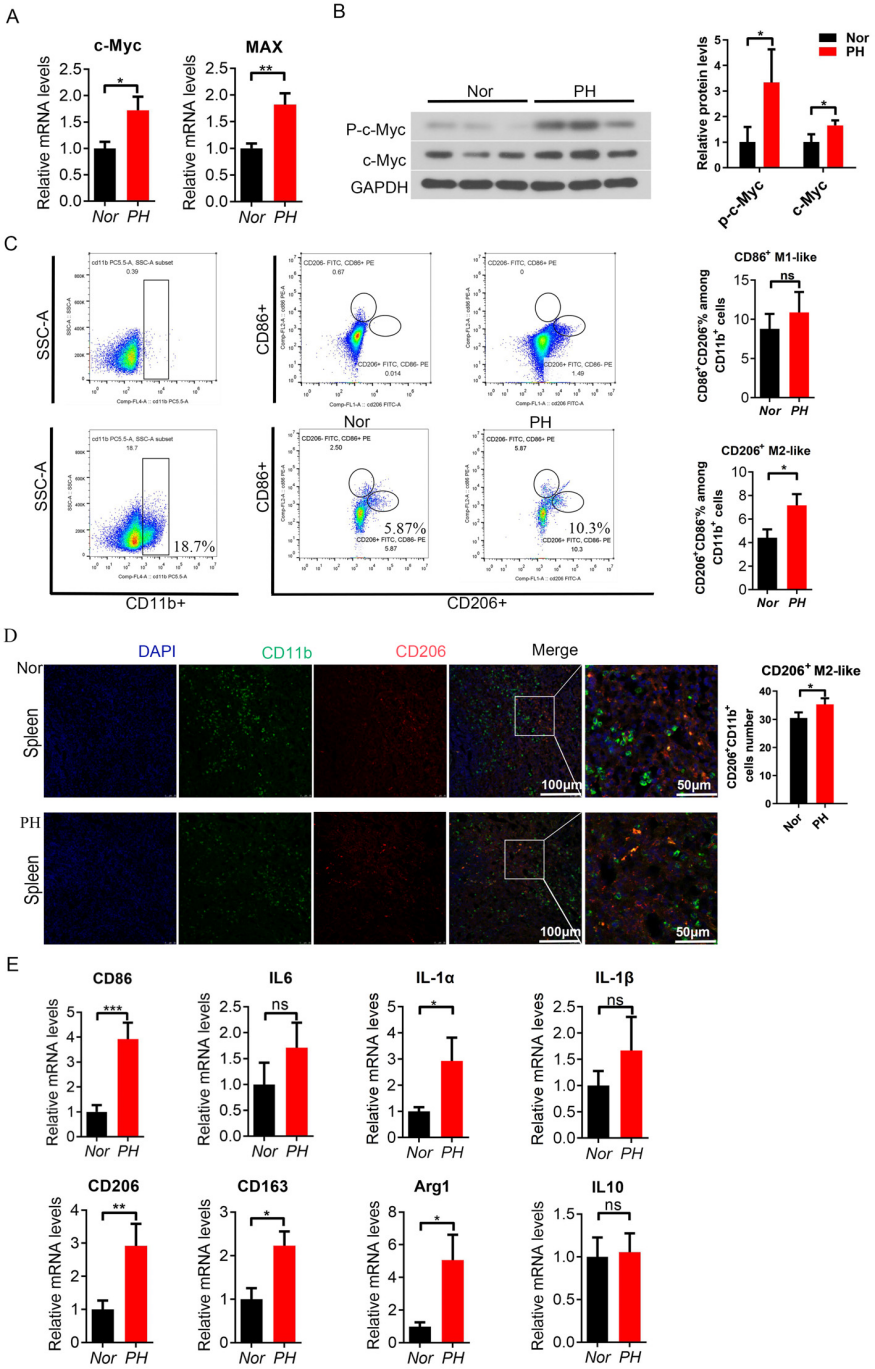
c-Myc increased and M2-like spleen macrophages were activated in PH patients. **(A)** c-Myc and Max mRNA expression in spleen macrophages sorted by magnetic beads (CD14^+^) in the Nor group and PH group, analyzed by qPCR (8 samples in the Nor group and 16 samples in the pH group). **(B)** p-c-Myc and c-Myc protein expressions were higher in PH macrophages than in Nor macrophages, as revealed by western blot (3 samples/group). **(C)** Ratio of CD86^+^ M1-like macrophages (CD11b^+^CD86^+^CD206^-^) and CD206^+^ M2-like macrophages (CD11b^+^CD86^-^CD206 ^+^) in splenocyte suspension analyzed by FACS, and the significant difference between the two groups was assessed (6 samples in the Nor group and 9 samples in the PH group). **(D)** Positive cells of CD206^+^ M2-like macrophages (CD11b^+^CD206^+^) in the spleen of patients, as detected by immunofluorescence double staining. **(E, F)** Relative mRNA expression of M1-like and M2-like macrophage related inflammatory factors in the spleen (8 samples in the Nor group and 16 samples in the PH group). *p<0.05, **p<0.01, ***p<0.001, ns p>0.05.

This study used CD14 magnetic bead sorting of monocyte-macrophages showed high specificity, and high-purity macrophages were obtained from patients with normal traumatic spleen and splenic hypersplenism (92.24% and 89.94%) (**Supplementary Figures S1A, B**). When using CD14 and CD11b antibodies to label and sort positive cells simultaneously, the proportion of CD14^+^ cells and CD11b^+^ cells was similar (90.2% vs 89%) (**Supplementary Figures S1A, B**). C-Myc and MAX mRNA expression in splenic macrophages was increased compared to that in normal traumatic spleen (**Figure 1A**). Similarly, c-Myc and phosphorylated c-Myc protein expression significantly increased (*P*<0.05, **Figure 1B**). Further FACS analysis revealed changes in the proportion of CD86^+^ M1-like macrophage and CD206^+^ M2-like macrophage in the spleen (PH *vs* Nor); the proportion of CD206^+^ M2-like macrophages (CD11b^+^CD86^-^CD206^+^) or (CD11b^+^CD11c^-^CD206^+^) in PH patients significantly increased (**Figure 1C**, **Supplementary Figure S1C**), while the change in CD86^+^ M1-like macrophages (CD11b^+^ CD86^+^ CD206^-^) or (CD11b^+^ CD11c^+^ CD206^-^) was not significant (**Figure 1C**, **Supplementary Figure S2C**). CD206^+^ M2-like macrophages (CD11b^+^ CD206^+^) showed an increasing trend in the PH spleen (*P* < 0.05, **Figure 1D**). The mRNA expression of the M1-like related inflammatory factors CD86, IL-6, IL-1α, and IL-1β showed an upward trend in splenic macrophages of PH patients (**Figure 1E**). The mRNA expression of M2-like inflammatory factors CD206, CD163, IL-10, and related ARG1 is significantly upregulated (*P* < 0.05, **Figure 1F**). These results demonstrated that the expression of the c-Myc gene was increased in patients with hypersplenism of PH; M2-like macrophages were significantly activated in patients with hypersplenism.

### 3.2 MAPK signaling pathway was activated in spleen macrophages of PH patients

**Figure 2:**
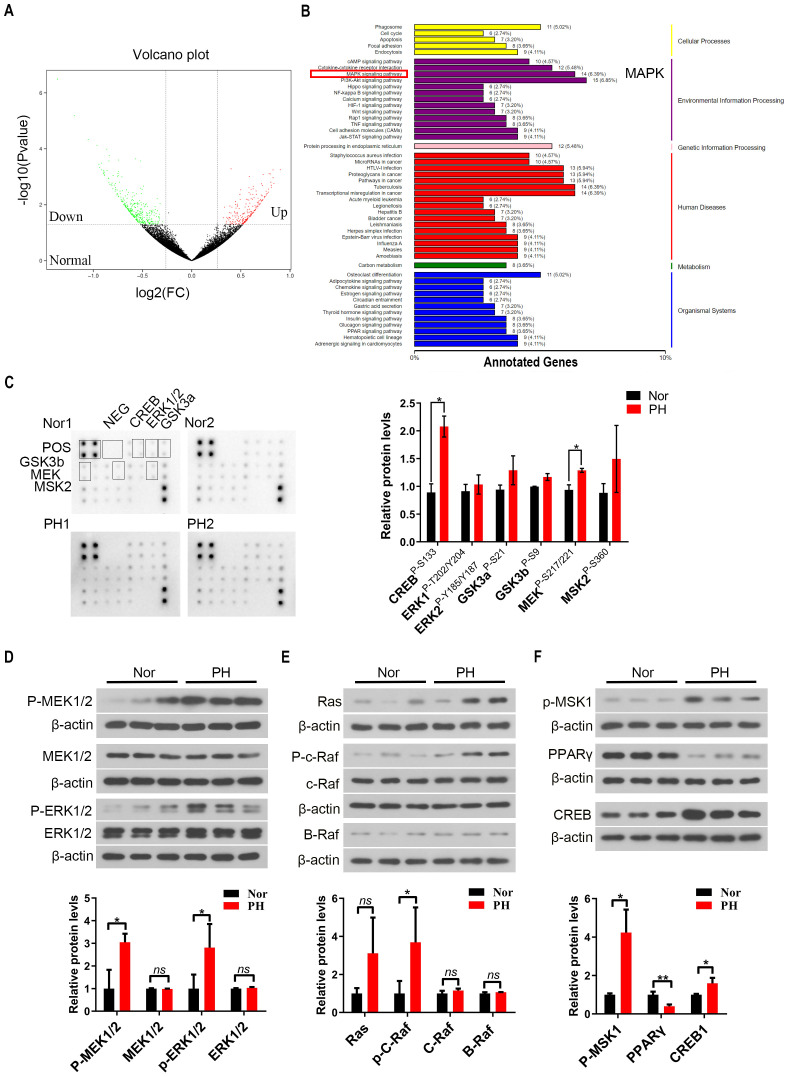
MAPK signaling pathway was activated in spleen macrophages of hypersplenism patients with portal hypertension (PH). **(A, B)** RNA-seq was performed on spleen macrophages (CD14^+^) in the Nor group and PH group (6 samples in each group), and the volcano plot showed the distribution of the differentially expressed genes (DEGs). KEGG enrichment analysis of RNA-seq data showing the expression of the related pathways in the Nor group *vs* the pH group. **(C)** Expression of MAPK pathway related proteins was further detected by MAPK pathway phosphorylation array (2 samples in each group), and the relative expression of CREB, ERK1/2, GSK3a/b, MEK, and MSK2 proteins in the Nor group *vs* PH group was assessed. **(D–F)** p-MEK1/2, MEK1/2, p-ERK1/2, ERK1/2, Ras, p-c-Raf, c-Raf, B-Raf, p-MSK1, PPARγ, and CREB protein expression in spleen macrophages of the Nor group and pH group (3 samples in each group) by western blot. β-actin was used as the loading control. A two-tailed Student *t*-test was used to examine the significance of each gene. Results are expressed as mean ± SD. Nor, normal traumatic spleen; PH, hypersplenism spleen; CREB, cAMP-response element binding protein; ERK1/2, extracellular regulated protein kinases1/2; GSK3a/b, glycogen synthase kinase3a/b; PPARγ, peroxisome proliferators-activated receptor. *p<0.05, ns p>0.05.

JAK-STAT and TLR4/NF-κB signaling pathways in the PH group were activated compared to the Nor group (**Supplementary Figures S2A, B**), consistent with the reported results in previous studies (16). KEGG analysis showed that MAPK signaling pathway was activated in patients with hypersplenism (**Figures 2A, B**). In addition, the MAPK signaling pathway was significantly activated, showing that the expression of MEK (P-S217/221), ERK1 (P-T202/Y203), and ERK2 (P-Y185/Y187) phosphorylation in macrophages of PH patients was significantly increased, as well as the expression of downstream phosphorylated MSK2 and the expression of CREB phosphorylation s133 site (*P* < 0.05) (**Figure 2C**, **Supplementary Figure S2C**). The expression of phosphorylated MEK1/2 and phosphorylated ERK1/2 in pH spleen macrophages significantly increased compared to that in normal spleen (*P* < 0.05). The expression of the upstream Ras, p-c-Raf, c-Raf, and B-Raf significantly increased as well as that of the downstream p-MSK1 and CREB (*P* < 0.05) (**Figures 2D–F**). Previous studies reported that the activity of PPARγ is inhibited by MAPK phosphorylation (26). The expression of PPARγ protein in macrophages was significantly reduced in the PH spleen (*P* < 0.01), further suggesting the activation of the MAPK pathway (**Figure 2F**). These results demonstrated that the MAPK signaling pathway in PH patients was activated through the Ras-Raf-MEK-ERK axis.

### 3.3 PH patients induce M2-like activation through the MEK-ERK-c-MYC axis

**Figure 3:**
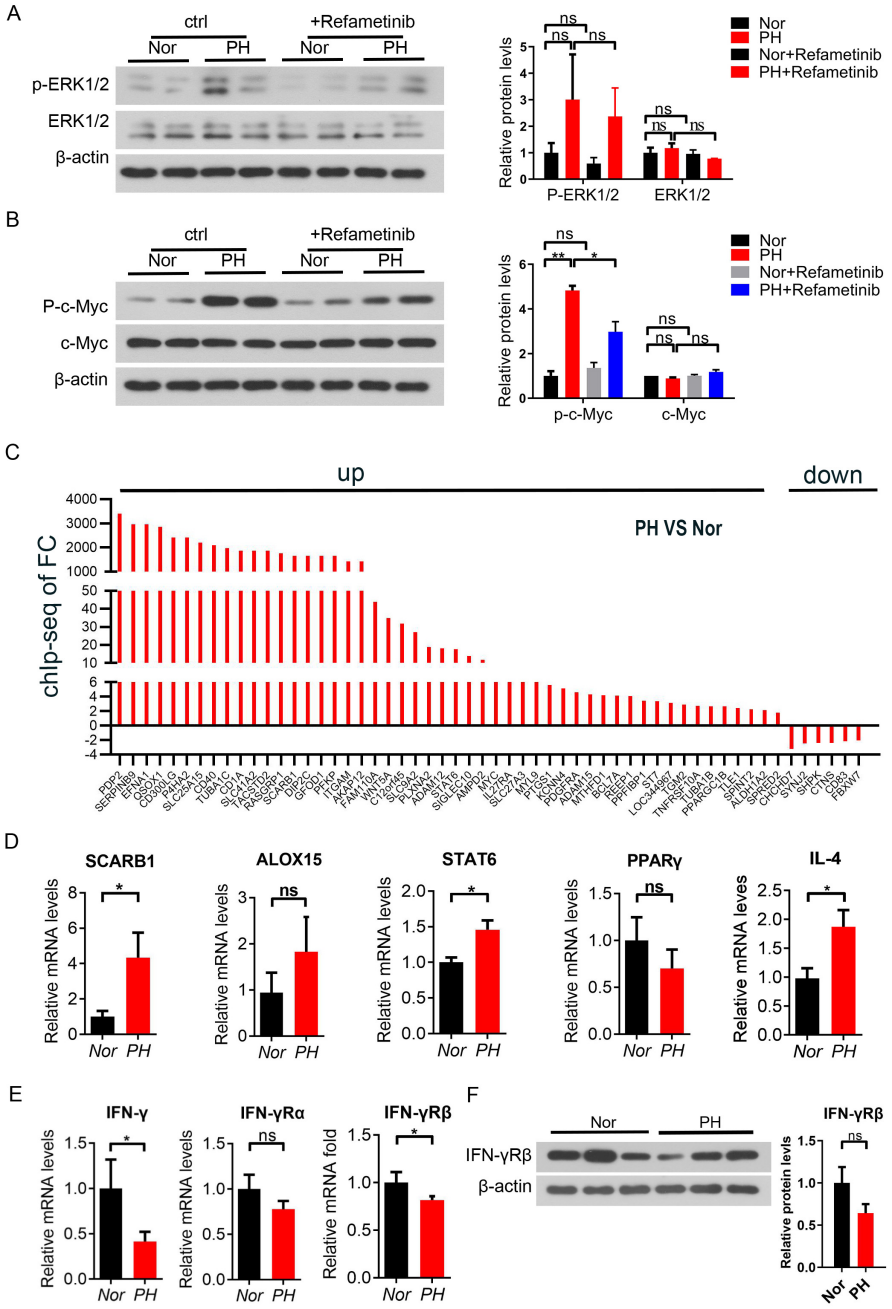
c-Myc participated in the inflammatory response of pH patients through the MEK/ERK signaling pathway. **(A, B)** p-MEK, MEK, p-c-Myc, and c-Myc protein expression in spleen macrophages of the Nor group and PH group by western blot after the *in vitro* culture and treatment with Refametinib (2 samples in each group). **(C)** Expression of c-Myc-dependent genes in PH *vs* Nor M2-like macrophages compared by immuno-coprecipitation analysis (chip) and chip seq detection. **(D)** SCARB1, ALOX15, STAT6, PPARγ and IL4 mRNA expression in PH *vs* Nor macrophages by qPCR **(E, F)** IFN-γ mRNA and protein expression in PH *vs* Nor macrophages by qPCR and western blot. *p<0.05, **p<0.01, ns p>0.05.

Further *in vitro* cytology experiments were conducted to add MEK inhibitors to the mononuclear cells of the patient’s spleen, and to investigate the activation of the MEK/ERK signaling pathway in macrophages. Use CCK8 to titrate the drug concentration of refametinib (MEK inhibitor) (**Supplementary Figure S3A**). At the same time, Trametinib, selumetinib, and refametinib had a significant inhibitory effect on MEK1/2 in macrophages, with a reduced downstream c-Myc expression. Among them, refametinib exerted the best inhibitory effect (**Supplementary Figures S3B, C**). The expression of phosphorylated ERK1/2 and phosphorylated c-Myc increased in macrophages of patients with hypersplenism compared with that in macrophages of Nor patients spleen, suggesting the activation of MAPK signaling pathway (**Figures 3A, B**). The expression of phosphorylated ERK1/2 and phosphorylated c-Myc in macrophages of the Nor group and hypersplenism spleen was decreased after the administration of the inhibitor compared with that in the macrophages of the control group. A significant difference was observed in the increase of phosphorylated c-Myc (*P* < 0.05) (**Figures 3A, B**). Based on these results, inhibiting the activation of the MAPK signaling pathway affects the expression of phosphorylated c-Myc protein in PH patients. Therefore, c-Myc is involved in the activation of the MAPK pathway in PH patients (**Figures 3A, B**). We further conducted a C-Myc ChIP studies on splenic macrophages and performed PCR quality controls and qPCR fold enrichment analysis on each Chip sample (**Supplementary Figure S4**). We also performed Chip-seq analysis on two samples. Compare the expression changes of c-Myc-dependent genes in PH and Nor M2 like macrophages. Among the 56 related genes analyzed, 51 genes showed an increased expression (**Figure 3C**). The expression of SCARB1, ALOX15, STAT6, and IL4 was up-regulated in hypersplenism, while the PPARγ expression was down-regulated (**Figure 3D**). Moreover, IFN-γ genes in patients with hypersplenism were downregulated at both molecular and protein levels, with significant differences (**Figures 3E, F**). The comprehensive results indicated that patients with hypersplenism showed an induction of macrophage M2-like activation through the MEK-ERK-c-MYC axis. c-Myc was involved in regulating the expression of alternative activation pathway-dependent genes, probably through the upregulation of IL-4-mediated signal transduction downregulating PPARγ and IFN-γ.

### 3.4 c-Myc increased and M2-like macrophages were activated in liver fibrosis mice

Using CD11b magnetic beads to sort mouse spleen Monocyte-macrophages, the results showed that CD11b has high specificity, and different treatment groups can obtain high-purity monocytes-macrophages(CD11b^+^) (**Supplementary Figure S5A**). When using CD11b and F4/80 antibodies to label and sort positive cells simultaneously, the proportion of CD11b^+^cells and F4/80^+^cells was similar (82.7% vs 83.7%), and the proportion of CD11b^+^F4/80^+^cells was 74.2% (**Supplementary Figure S5B**) This result suggests that mouse spleen macrophages account for a high proportion of positive cells sorted by CD11b magnetic beads, which can be used for subsequent research.

**Figure 4:**
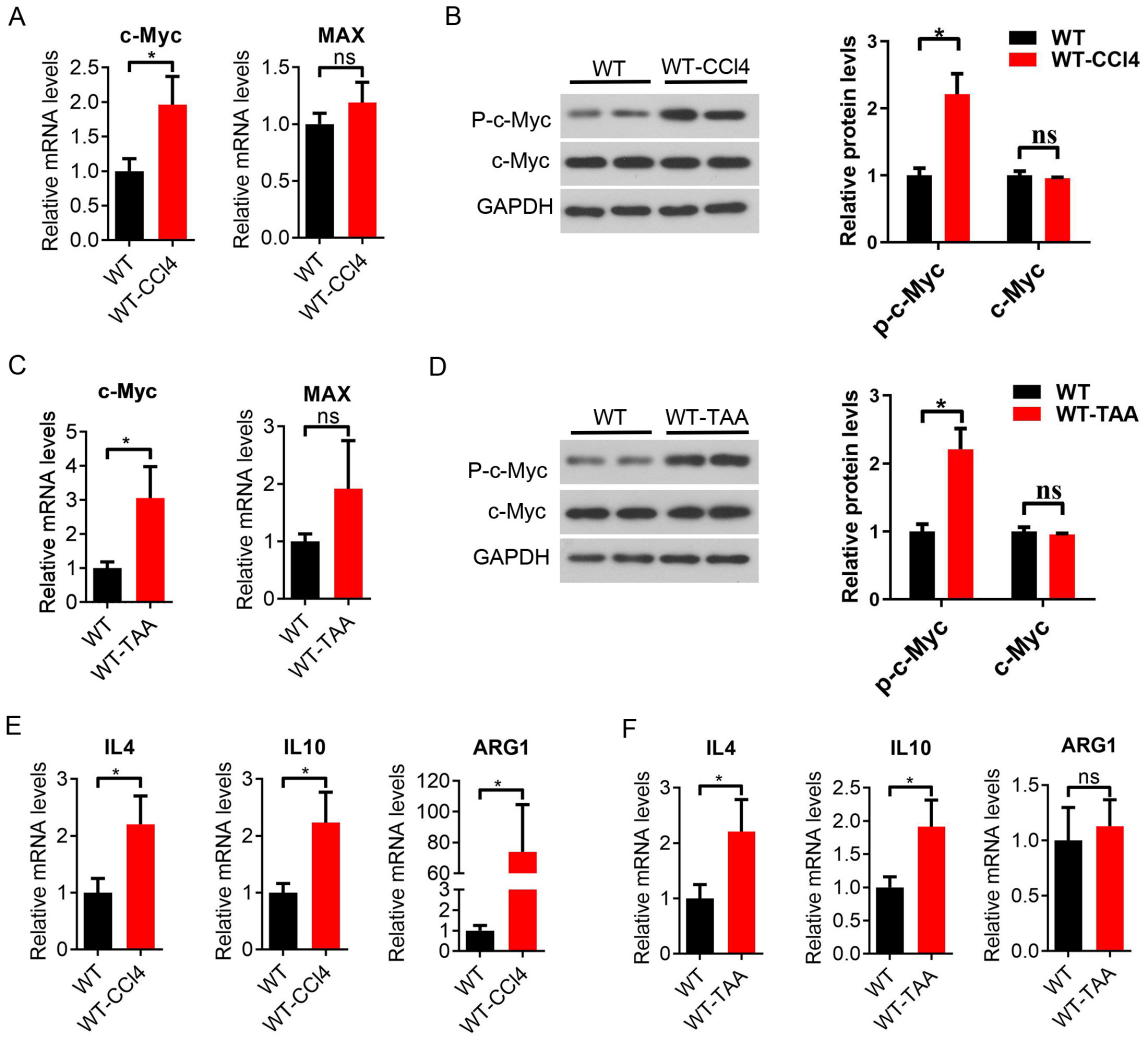
Increased c-Myc expression and activation of M2-like macrophages in CCl4 and TAA-induced liver fibrosis mice. **(A)** Expression of CD11b ^+^ c-Myc and Max mRNA in mouse spleen macrophages sorted by magnetic beads (CD11b ^+^) in the WT group and WT-CCl4 group, analyzed by qPCR (6 samples in the WT group and 5 samples in the WT-CCl4 group). **(B)** p-c-Myc and c-Myc protein expression in spleen macrophages of the WT group and WT-CCl4 group (2 samples in each group). **(C)** c-Myc and Max mRNA expression in spleen macrophages of the WT group and WT-TAA group (6 samples in each group). **(D)** p-c-Myc and c-Myc protein expression in spleen macrophages of the WT group and WT-TAA group by western blot (2 samples in each group). **(E, F)** Spleen M2-like macrophage-related inflammatory factors IL4, IL10, Arg1 mRNA expression in the CCl4 and TAA model group by qPCR (6 samples in each group). *p<0.05, ns p>0.05.

In addition, the expression of c-Myc and Max mRNA was upregulated in the spleen macrophages of CCl4-induced liver fibrosis mice (**Figure 4A**), and the expression of phosphorylated c-Myc protein was increased in the spleen macrophages of the model mice compared with the control group (*P* < 0.05, **Figure 4B**). The expression of c-Myc and Max mRNA was upregulated in the spleen macrophages of TAA-induced liver fibrosis mice, and the Max difference is not significant (**Figure 4C**), and the expression of phosphorylated c-Myc protein was increased in the spleen macrophages of TAA model mice (*P* < 0.05, **Figure 4D**). The mRNA expression of the M2-related inflammatory factors IL4, IL10 and Arg1 was significantly upregulated in spleen macrophages in CCl4 model mice (*P* < 0.05, **Figure 4E**). The mRNA expression of the M2-like related inflammatory factors IL4, IL10 and arg1 in TAA model mice increased in spleen macrophages, with a significant difference between IL4 and IL10 (*P* < 0.05) (**Figure 4F**). These results suggested that the increased expression of the c-Myc gene in spleen macrophages of CCl4 and TAA-induced hepatic fibrosis mice might be involved in hepatic fibrosis inflammation and M2-like macrophage activation in hepatic fibrosis mice.

### 3.5 Loss of c-Myc in macrophages aggravated CCl4 and TAA induced hepatic fibrosis and inflammation

**Figure 5:**
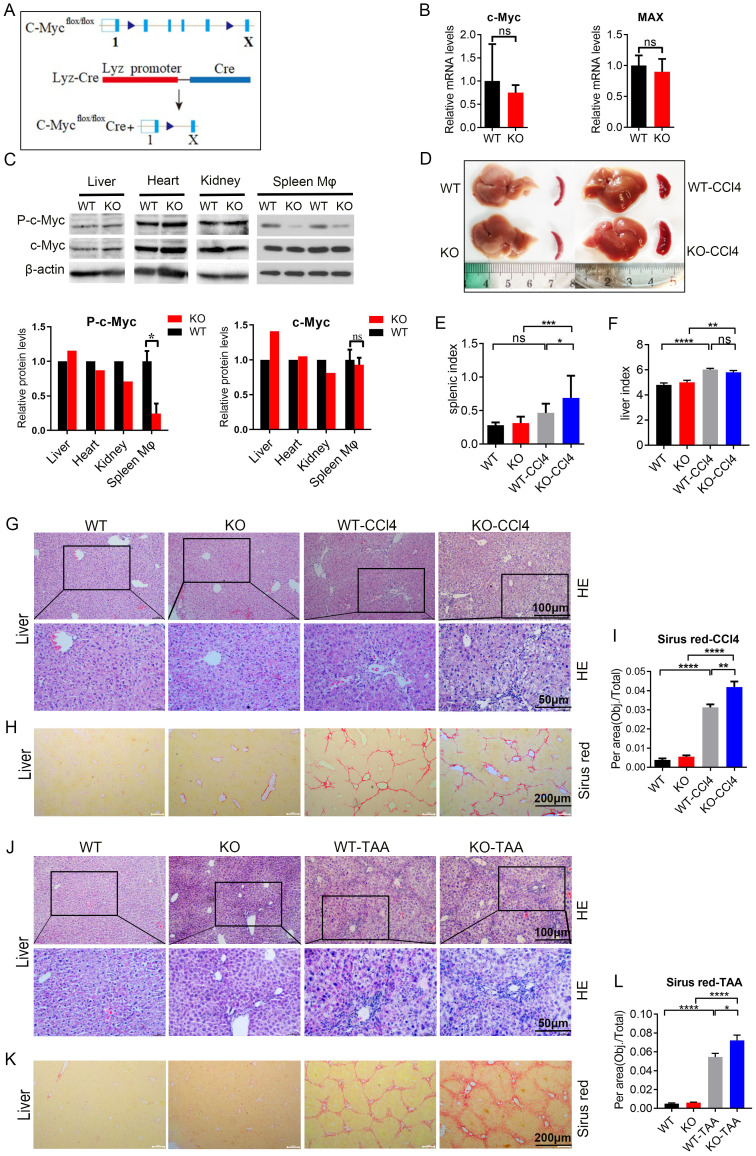
c-Myc deletion in macrophages aggravated CCl4 and TAA-induced liver fibrosis and inflammation **(A)** Protocol to obtain macrophage specific c-Myc knockout mice. **(B)** c-Myc and Max mRNA expression in spleen macrophages of knockout mice by qPCR (5 samples in each group). **(C)** p-c-Myc and c-Myc protein expressions in the liver, heart, kidney, and spleen macrophages of WT and KO mice analyzed and compared by western blot. **(D–F)** Liver and spleen morphology, spleen index, and liver index in the knockout group and model mice of the CCl4 induced liver fibrosis model compared to the control group (9 samples in WT and KO groups, 17 samples in WT-CCl4 group and 12 samples in KO-CCl4 group) **(G, H)**. In the CCl4 induced liver fibrosis model, the pathological changes of the liver were analyzed by HE staining, and the degree of liver fibrosis was evaluated by Sirus red staining. **(I)** Quantification of the fibrosis. **(J–L)** TAA-induced liver fibrosis model, as shown by with HE staining and Sirus red staining, and quantification of the fibrosis (5 samples in WT and KO groups, 9 samples in WT-TAA group, and 10 samples in KO-TAA group). * p<0.05, ** p<0.01, *** p<0.001, **** p<0.0001, ns p>0.05.

c-Myc and Max mRNA expression in the KO group decreased compared with that in the WT group (**Figures 5A, B**). p-c-Myc protein expression in spleen macrophages was significantly lower than that in liver, heart, and kidney macrophages (*P* < 0.05, **Figure 5C**). c-Myc protein expression was not significantly decreased, which was related to the splicing position of exon (**Figure 5C**), In this mice model (Lyz cre^+/-^ c-Myc loxp^+/-^) the C-Myc gene in KO mice was specifically knocked out in macrophages.

Compared with the control group, both CCL4 and TAA models showed an increase in liver index and spleen index in mice, with a significant difference in spleen index (P<0.05) (**Figures 5D–F**; **Supplementary Figures S6A–C**). At the same time, research found that the liver index and spleen index of KO group mice further increased under modeling conditions, with significant differences (P<0.01) (WT-CCL4 vs KO KO-CCL4; WT-TAA vs KO-TAA) (**Figures 5D-F**; **Supplementary Figures S6A–C**). These results suggest that the liver and spleen of mice increased after modeling, while the liver and spleen of C-Myc KO mice showed an increasing trend of enlargement.

The liver of CCl4 and TTA model mice showed evident inflammatory cell infiltration and collagen fiber proliferation than the control group; c-Myc KO model mice showed more severe liver inflammation, and the spleen of the model mice showed evident disorder of splenosome marginal area (WT-CCL4 vs KO KO-CCL4; WT-TAA vs KO-TAA) (**Figures 5G, J**; **Supplementary Figure S6D**). The liver of CCl4 and TTA model mice showed more evident liver fibrosis than the control group, and the liver fibrosis in the c-Myc KO model mice (KO-CCl4 and KO-TAA) was aggravated (**Figures 5H, K**; **Supplementary Figure S6E**). These results showed that liver fibrosis and spleen inflammation in spleen macrophage-specific c-Myc knockout mice were aggravated in the liver fibrosis model induced by CCL4 and TAA.

### 3.6 c-myc gene regulated the polarization of macrophages to M2-like in a mouse model of liver fibrosis

**Figure 6:**
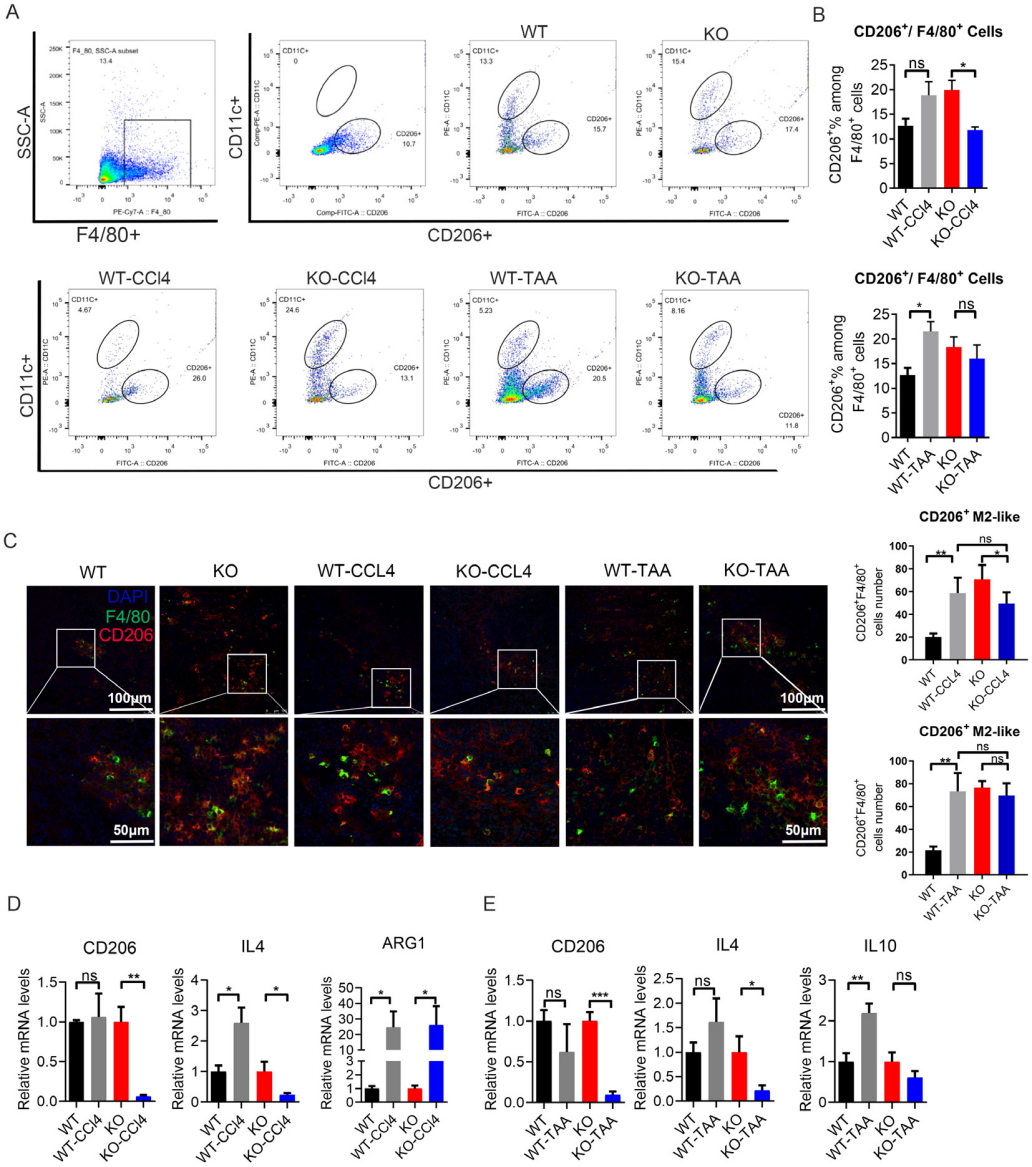
c-Myc deletion in macrophages reduced the polarization of M2-like macrophages in mice with liver fibrosis. **(A, B)** Proportion of spleen CD206^+^ M2-like macrophages (F4/80 ^+^ CD11c^-^ CD206^+^) in the WT and KO group after CCl4 and TAA administration by FACS (5 samples in each group). **(C)** Positive cells of CD206^+^ M2-like macrophages (F4/80^+^CD206^+^) in the spleen of mouse, as detected by immunofluorescence double staining. **(D, E)** Relative mRNA expression of M2-like macrophage-related inflammatory factors CD206, IL4, IL10, and Arg1 in different groups after CCl4 and TAA modeling, by qPCR (5 samples in each group). *p<0.05, **p<0.01, ***p<0.001, ns p>0.05.

The proportion of CD206^+^ M2-like macrophages (F4/80^+^ CD11c^-^ CD206^+^) in the WT-CCL4 and WT-TAA group increased compared with that in the WT group mice, with a significant difference in the CCL4 model group (WT *vs* WT-CCl4), *P* < 0.05 (**Figures 6A, B**). The proportion of CD206^+^ M2-like type macrophages (F4/80^+^ CD11c^-^ CD206^+^) in the KO-CCL4 and KO-TAA group decreased compared with that in the KO group, with a significant difference in the KO-CCl4 group (KO *vs* KO-CCl4), *P* < 0.05 (**Figures 6A, B**). The proportion of total macrophages (F4/80^+^) and CD11c^+^ M1-like macrophages (F4/80^+^ CD11C^+^ CD206^-^) decreased compared to that in the KO group (**Supplementary Figure S7**). CD206^+^ M2-like macrophages (F4/80^+^/CD206^+^) showed an increasing trend in the PH spleen (*P* < 0.05, **Figure 1D**). In histology, the proportion of CD206+M2 like macrophages (F4/80+/CD206+) showed an increasing trend in liver fibrosis mice (WT vs WT-CCL4, WT VS WT-TAA), P<0.05, **Figure 1D**). In macrophage c-Myc knockout mice, the proportion of CD206+M2 like macrophages in the modeling group showed a downward trend (KO vs KO-CCL4, KO vs KO-TAA), with significant differences observed in the CCL4 modeling group (P<0.05, **Figure 1D**). Moreover, the mRNA expression of the M2-like related inflammatory factors CD206 and IL-4 in the KO-CCL4 group significantly decreased compared with that in the KO group (*P* < 0.05) (**Figure 6D**). The mRNA expression of the M2-like related inflammatory factors CD206, IL-4 and IL-10 in the KO-TAA group was down-regulated compared with that in the KO group, and the down-regulation of the CD206 gene was significant (**Figure 6E**). These results showed that c-Myc deletion reduced the proportion and polarization of M2-like macrophages in CCL4 and TAA-induced liver fibrosis model, and c-Myc gene regulated the polarization of M2-like macrophages.

### 3.7 C-Myc deletion inhibited the activation of MAPK signaling pathway

**Figure 7:**
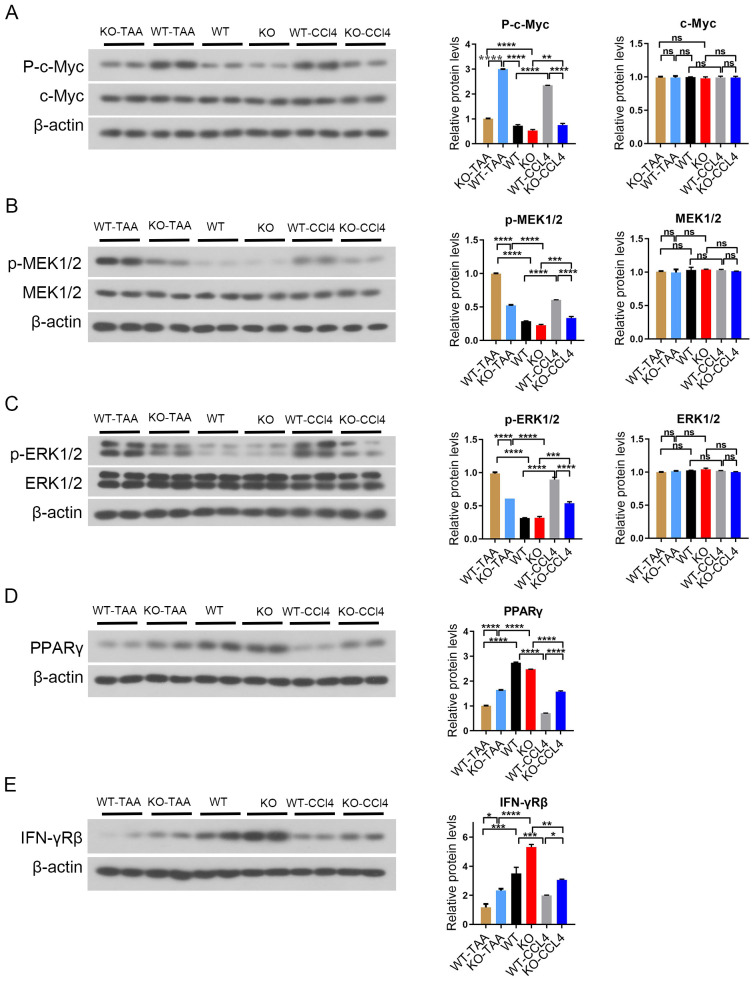
c-Myc deletion in spleen macrophages reduced the activation of MAPK signaling pathway. **(A)** p-c-Myc and c-Myc protein expression in splenic macrophages in each group, and the significant difference among the groups was calculated (2 samples in each group). **(B, C)** p-MEK1/2, MEK1/2, p-ERK1/2, and ERK1/2 protein expression in splenic macrophages in each group, and the significant difference among the groups was calculated (2 samples in each group). **(C)** MEK pathway inhibition. **(D)** Downstream PPARγ gene protein expression and significant difference between groups (2 samples in each group). **(E)** IFN-γRβ protein expression in splenic macrophages in each group. * p<0.05, ** p<0.01, *** p<0.001, **** p<0.0001, ns p>0.05.

The expression of the phosphorylated c-Myc in spleen macrophages of WT-CCL4 mice was significantly higher than that of WT mouse. The expression of phosphorylated c-Myc significantly decreased in the KO-CCL4 group compared with that in the WT-CCL4 group (WT-CCL4 *vs* KO-CCL4, *P* < 0.001) (**Figure 7A**). The expression of phosphorylated MEK1/2 and phosphorylated ERK1/2 in spleen macrophages of the WT-CCL4 group mice significantly increased compared with that in the spleen macrophages of the WT group. The expression of phosphorylated MEK1/2 and phosphorylated ERK1/2 significantly decreased in the KO-CCL4 group compared with that in the WT-CCL4 group (*P* < 0.001) (**Figures 7B, C**). Mouse spleen macrophages PPARγ gene expression was downregulated in the CCL4 group (WT *vs* KO-CCL4). c-Myc gene knockout significantly upregulated PPARγ expression (WT-CCL4 *vs* KO-CCL4) (*P* < 0.001) (**Figure 7D**). IFN-γRβ expression increased in the KO group mice compared with that in the WT group mice. IFN-γRβ expression in CCL4 and TAA model group was significantly reduced compared with that in the control group (WT-CCL4 *vs* WT, KO-CCL4 *vs* KO) (*P* < 0.01) (**Figure 7E**). The MEK/ERK signaling pathway was activated in spleen macrophages of CCl4 or TAA-induced liver fibrosis mice. The c-Myc gene feedback regulated the activation of MAPK signaling pathway and regulated the PPARγ and IFN-γ expression.

## 4 Discussion

Hypersplenic function is a common manifestation of liver cirrhosis, which leads to a decrease in the number of third line cells and induces upper gastrointestinal bleeding, posing a serious threat to the patient’s life. Splenectomy is usually performed on patients with liver cirrhosis and hepatitis, which improves immune response, and reduces the risk of liver cancer (27–29). However, still many complications can occur; splenectomy increases the risk of portal vein thrombosis in patients with liver cirrhosis by at least 10 times (30). Our previous studies revealed that macrophage phagocytose blood cells in the spleen of patients with splenomegaly lead to the abnormal activation of splenic macrophages. However, the type and mechanism of activation are unclear.

Splenectomy regulates macrophages phenotypic conversion in hepatic fibrosis. promotes fibrosis and fibrosis ablation in the progression and regression of liver cirrhosis, respectively (21, 31, 32). The study found that macrophages are recruited to the tissue injury area at different stages of liver fibrosis, and they switch between different states, including pro-inflammatory M1 and anti-inflammatory M2, affecting liver fibrosis through phenotypic transformation (12, 33–35). Knockout of mir-98-5p alleviates inflammatory bowel disease symptoms by increasing the expression of Trib1 and changing the polarization of macrophages to M2 phenotype (36). In kidney injury inflammation and fibrosis, macrophages differentiate into M2-like by releasing IL10, arginase, TGF-β, and HO-1 to play an anti-inflammatory role (12, 37). The results of this study also confirmed that M2-like macrophages were activated in patients with hypersplenism, and the inhibition of M2-like polarization of macrophages led to increased inflammation of liver fibrosis in mice. Therefore, the intervention on M2-like polarization has become an important approach of alleviating liver inflammation and splenomegaly.

MYC produces the transcription factor Myc, which dimerizes with Max and binds target DNA sequences or E boxes (with the sequence 50-CANNTG-30) to regulate the transcription of genes involved in cell growth and proliferation (38). Multiple studies reported that the c-Myc gene is involved in regulating the inflammatory response of M2 macrophage (14, 39–41). In this study, the expression of c-Myc in splenic macrophages was upregulated in patients with hypersplenism and in two types of liver fibrosis model mice. Macrophage c-Myc specific knockout inhibited M2-like polarization and affected liver fibrosis inflammation. The c-Myc gene in splenic hypersplenism might be a transcription factor of M2-like macrophages, participating in the regulation of macrophages in patients with PH splenic hypersplenism. Therefore, regulating the expression of the c-Myc gene might alter the inflammation and microenvironment of patients with hypersplenism.

ERKs can not only phosphorylate envelope proteins, but also some nuclear transcription factors, such as c-fos, c-jun, c-Myc, and ATF2, which are involved in the regulation of cell proliferation and differentiation (42). c-Myc is a downstream effector of ERK signaling, and the central metabolic axis of MEK-ERK-c-Myc is also crucial in combating tumor progression (43). There are also many reports revealing that downstream c-Myc genes are involved in the regulation of negative feedback pathways in tumors (44, 45). It has been reported that ERK also phosphorylates the upstream proteins of its pathway, including neg receptor, SOS, Raf-1, and MEK, and then regulates the signaling pathway by its own negative feedback, participating in tumor regulation (46). This study confirmed the activation of the MEK/ERK signaling pathway in splenic macrophages in both hypersplenism patients and liver fibrosis mice. c-Myc is a downstream transcription factor of the MEK/ERK pathway. Patient samples showed the inhibition of MEK pathway activation and the downregulation of c-Myc gene expression. In liver fibrosis mice, c-Myc gene knockout reduced the activation of the MEK/ERK pathway; thus, c-Myc might participate in the polarization of M2-like macrophages through a feedback regulation of MAPK signaling pathway activation.

Previous studies showed that IL4 induces the activation of M2-like macrophages through different pathways (14, 47, 48). c-Myc directly regulates and replaces activation related genes, upregulates signaling mediators involved in IL4, and transcriptional activators STAS6 and PPARγ involved in the expression of tumor related macrophages (14). Research reports that IL-4-induced and MEK/ERK-mediated PPARγ and retinoic acid (RA) signaling are required for M2-like macrophage polarization (48). In this study, the results of patients with hypersplenism showed that the c-Myc regulatory alternative activation pathway dependent genes by upregulation of IL-4 mediated signal transduction to participate in M2-like polarization and downregulation of PPARγ and IFN-γ gene expression. Animal experiments showed that c-Myc knockout led to a decrease in macrophage IL-4 expression and increase PPARγ activation, while the expression of IFN-γRβ was suppressed, further supporting the results obtained with patients. The specific mechanism of IFN-γ And PPARγ involved in the polarization of M2-like macrophages still needs further studies in patients with PH.

**Figure 8:**
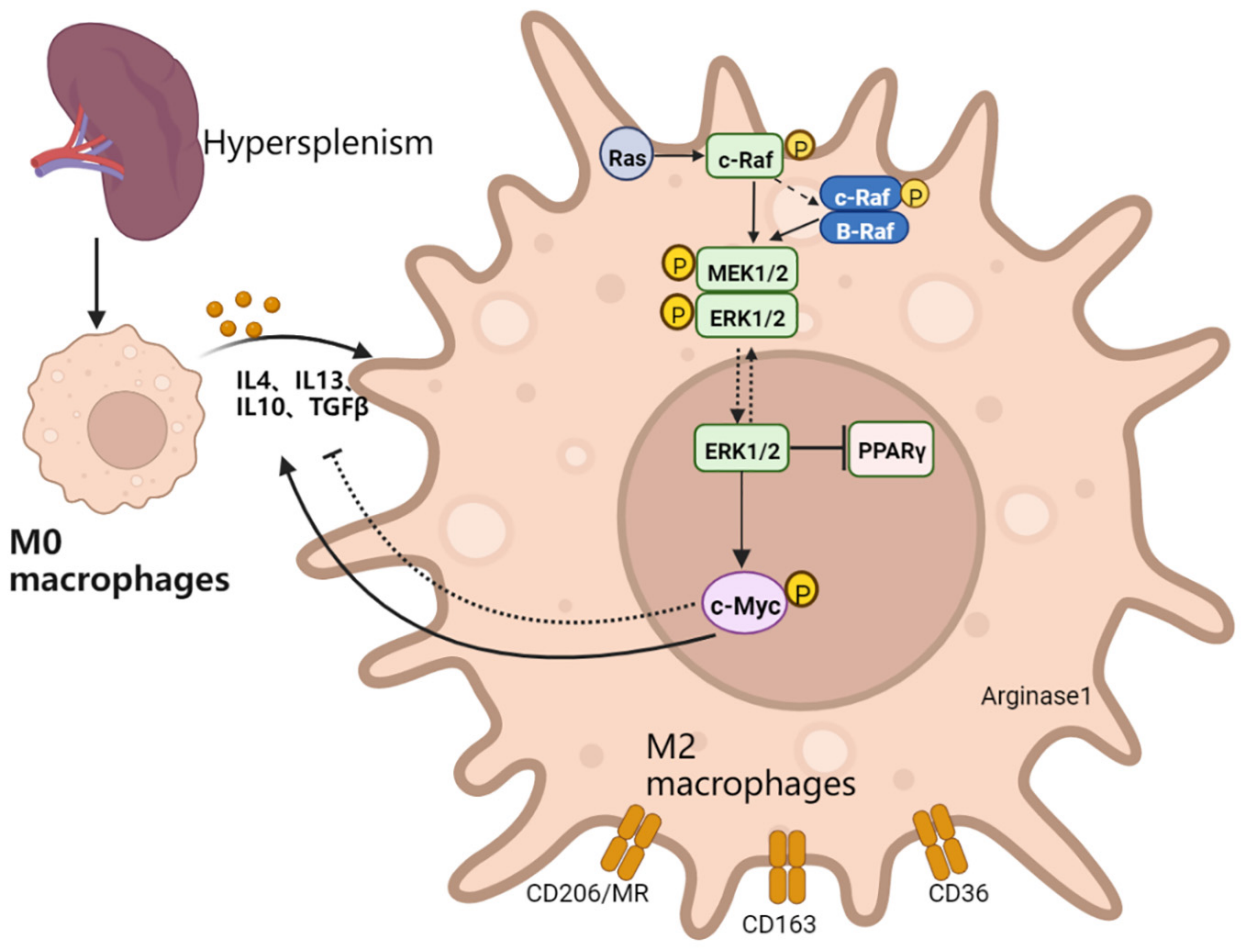
Schematic explanation of the mechanism of action: the transformation into M2-like of spleen macrophages in patients with PH is activated, which in turn activates the Ras-Raf-MEK-ERK-c-Myc signaling pathway axis. The inhibition of the MEK signaling pathway reduces the expression of c-Myc. Macrophage c-Myc specific knockout inhibits macrophage activation into the M2-like, reduces repair ability, and exacerbates fibrotic liver inflammation. c-Myc might provide feedback on the activation of MAPK signaling pathway and upregulate the expression of IL4 and M2-like related genes.

In conclusion, c-Myc regulates the activation of M2-like macrophages through the MEK-ERK-c-Myc axis in patients with hypersplenism. The c-Myc gene may exert anti-inflammatory effects by upregulating IL-4-mediated signal transduction to promote M2-like differentiation and anti-inflammatory cytokine secretion (**Figure 8**). Therefore, c-Myc in macrophages may become an important target for polarization therapy.

## Data Availability

The datasets presented in this study can be found in online repositories. The names of the repository/repositories and accession number(s) can be found below:GSE264420 and GSE264544 (GEO).
