# Supplementary material for: Activation of MEK‐ERK‐c‐MYC signaling pathway promotes splenic M2-like macrophage polarization to inhibit PHcH-liver cirrhosis
Source: Front Immunol. 2024 Nov 8;15:1417521. doi: 10.3389/fimmu.2024.1417521 (PMC11605246; doi:10.3389/fimmu.2024.1417521)
Supplement: Supplementary file 1 [file DataSheet1.docx]

**Supplementary information**

**Supplementary Table 1. Antibodies used** **in flow cytometry.**

| **Antibody** | **Conjugate** | **Clone** | **Isotype** | **Manufacturer** |
| --- | --- | --- | --- | --- |
| Anti-human  CD14 | PE/Cyanine 7 | HCD14 | Mouse IgG1 | Biolegend, Cat. No. 325618 |
| Anti- mouse /human CD11b | APC | M1/70 | Rat IgG2b | Biolegend, Cat. No. 101212 |
| Anti- mouse /human CD11b | PerCP/Cyanine5.5 | M1/70 | Rat IgG2b | Biolegend, Cat. No. 101227 |
| Anti-human  CD11c | PE | 3.9 | Mouse IgG1 | Biolegend, Cat. No. 301605 |
| Anti-human  CD206 | FITC | 15-2 | Mouse IgG1 | Biolegend, Cat. No. 321103 |
| Anti-human  CD86 | PE | BU63 | Mouse IgG1，K | Biolegend, Cat. No. 374205 |
| Anti- mouse /human CD11b | FITC | M1/70 | Rat IgG2b | Biolegend, Cat. No. 101205 |
| anti-mouse  F4/80 | PE/Cyanine 7 | BM8 | Rat / IgG2a | Biolegend, Cat. No. 123113 |
| anti­-mouse  CD11c | PE | N418 | Armenian Hamster IgG | Biolegend, Cat. No. 117307 |
| anti-mouse  CD206 | FITC | C068C2 | Rat / IgG2a | Biolegend, Cat. No. 141703 |

**Supplementary Table 2. Antibodies used** **in Western blot**

| Antibody | Cat No. | Manufacturer | KDa | [Dilute proportion](file:///C:\Program%20Files\baidu-translate-client\resources\app.asar\app.html) |
| --- | --- | --- | --- | --- |
| c-Myc | #13987 | CST | 57-65 | 1:1000 |
| p-C-Myc | #13748 | CST | 62 | 1:1000 |
| p-MEK1/2 | #9154 | CST | 45 | 1:5000 |
| MEK1/2 | #8727 | CST | 45 | 1:5000 |
| P-ERK1/2 | #4370 | CST | 44,42 | 1:5000 |
| ERK1/2 | #9102 | CST | 44,42 | 1:5000 |
| Ras | Ab52939 | abcam | 21 | 1:5000 |
| p-c-Raf | Ab157201 | abcam | 73 | 1:2000 |
| c-Raf | #9422 | CST | 65-75 | 1:3000 |
| B-Raf | #9433 | CST | 86 | 1:2000 |
| p-MSK1 | Ab81294 | abcam | 90 | 1:5000 |
| PPARγ | WL1800 | WanleiBio | 54/57 | 1:1000 |
| CREB | WL01848 | Wanleibio | 43 | 1:1000 |
| IFN-γRβ | Ab77246 | Abcam | 49 | 1:3000 |
| p-JAK2 | #3771 | CST | 125 | 1:1500 |
| JAK2 | #3230 | CST | 125 | 1:1500 |
| P-STAT1 | #9167 | CST | 91/84 | 1:1500 |
| STAT1 | #14994 | CST | 91/84 | 1:1500 |
| P-STAT3 | WLP2412 | WanleiBio | 91 | 1:400 |
| STAT3 | WL03207 | WanleiBio | 91 | 1:400 |
| TLR4 | WL00196 | WanleiBio | 120 | 1:400 |
| P-p65 | WL02169 | WanleiBio | 65 | 1:500 |
| p65 | WL01980 | WanleiBio | 65 | 1:1000 |
| GAPGH | ab181602 | abcam | 37 | 1:10000 |
| β-actin | WL01372 | Wanleibio | 42 | 1:1000 |

**Supplementary Table 3: Primers for real time-qPCR**

| Genes in human | |
| --- | --- |
| c-Myc | F TCCCTCCACTCGGAAGGAC |
|  | R CTGGTGCATTTTCGGTTGTTG |
| MAX | F TGGAGAAGGCGAGGTCAAGT |
|  | R CCCCATCGAAGGCAGAGAT |
| CD86 | F CTGCTCATCTATACACGGTTACC |
|  | R GGAAACGTCGTACAGTTCTGTG |
| IL6 | F ACTCACCTCTTCAGAACGAATTG |
|  | R CCATCTTTGGAAGGTTCAGGTTG |
| IL-1α | F TGGTAGTAGCAACCAACGGGA |
|  | R ACTTTGATTGAGGGCGTCATTC |
| IL-lβ | F AGCTACGAATCTCCGACCAC |
|  | R CGTTATCCCATGTGTCGAAGAA |
| CD206 | F GGGTTGCTATCACTCTCTATGC |
|  | R TTTCTTGTCTGTTGCCGTAGTT |
| CD163 | F GACGCATTTGGATGGATCATGT |
|  | R CCCACCGTCCTTGGAATTTGA |
| IL4 | F ATGGGTCTCACCTCCCAACT |
|  | R GATGTCTGTTACGGTCAACTCG |
| IL10 | F GACTTTAAGGGTTACCTGGGTTG |
|  | R TCACATGCGCCTTGATGTCTG |
| HK2 | F GCCCCGCAGGTAGTCAGG |
|  | R AGCCACGATTCTCTCCACG |
| PER1 | F AAAGAGGCGCAGCGTATCTC |
|  | R TTGCATAATGCCGGGCACTG |
| CRY1 | F GGCACCTCACGTTTCTGAAG |
|  | R GTGGACGAGGGTCAACAAGT |
| NCL | F TTTTGCGACGCGTACGAG |
|  | R ACTAGGGCCGATACCGCC |
| SCARB | F TGCAGTGTTTCACCTTGCAT |
|  | R GCCTCGGAAAACAACTTCTG |
| ALOX15 | F CTTGCTCTGACCACACCAGA |
|  | R GCTGGGGCCAAACTATATGA |
| STAT6 | F ATGGGGCAACAGAAAAGATG |
|  | R GCACAGAAGACAGCAGCAAG |
| PPARγ | F GGGCTCCATAAAGTCACCAA |
|  | R GCTGTGCAGGAGATCACAGA |
| IFN-γ | F TCGGTAACTGACTTGAATGTCCA |
|  | R TCGCTTCCCTGTTTTAGCTGC |
| IFN-γRα | F AGCAGGAAGTCGATTATGATCCC |
|  | R CTGGCACTGAATCTCGTCACA |
| IFN-γRβ | F AGCGATTCCAGTATCCTCACT |
|  | R CCAGGCTAAGCACTAGAAAGAGT |
| GAPDH | F CCCTTCATTGACCTCAACTACATG |
|  | R TGGGATTTCCATTGATGACAAGC |

| Genes in Mice | |
| --- | --- |
| c-Myc | F ATGCCCCTCAACGTGAACTTC |
|  | R CGCAACATAGGATGGAGAGCA |
| MAX | F ACCATAATGCACTGGAACGAAA |
|  | R GTCCCGCAAACTGTGAAAGC |
| CD206 | F CTCTGTTCAGCTATTGGACGC |
|  | R CGGAATTTCTGGGATTCAGCTTC |
| IL4 | F GGTCTCAACCCCCAGCTAGT |
|  | R GCCGATGATCTCTCTCAAGTGAT |
| IL10 | F GCTCTTACTGACTGGCATGAG |
|  | R CGCAGCTCTAGGAGCATGTG |
| GAPDH | F AGGTCGGTGTGAACGGATTTG |
|  | R TGTAGACCATGTAGTTGAGGTCA |
| Lyz cre | Mutant primer CCCAGAAATGCCAGATTACG |
|  | Common Primer CTTGGGCTGCCAGAATTTCTC |
|  | Wild Type Primer TTACAGTCGGCCAGGCTGAC |
| c-Myc | WT Primer GCCCCTGAATTGCTAGGAAGACTG |
|  | Delta Primer CCGACCGGGTCCGAGTCCCTATT |

Validated primers

|  | Gene | Cat No. | Manufacturer | Registration number |
| --- | --- | --- | --- | --- |
| Human | ARG1 | HQP010127 | GeneCopoeia | NM_000045.2 |
| Human | GAPDH | HQP006940 | GeneCopoeia | NM_002046.3 |
| Mice | ARG1 | MQP026580 | GeneCopoeia | NM_007482.3 |

1. **Supplementary Figure 1**


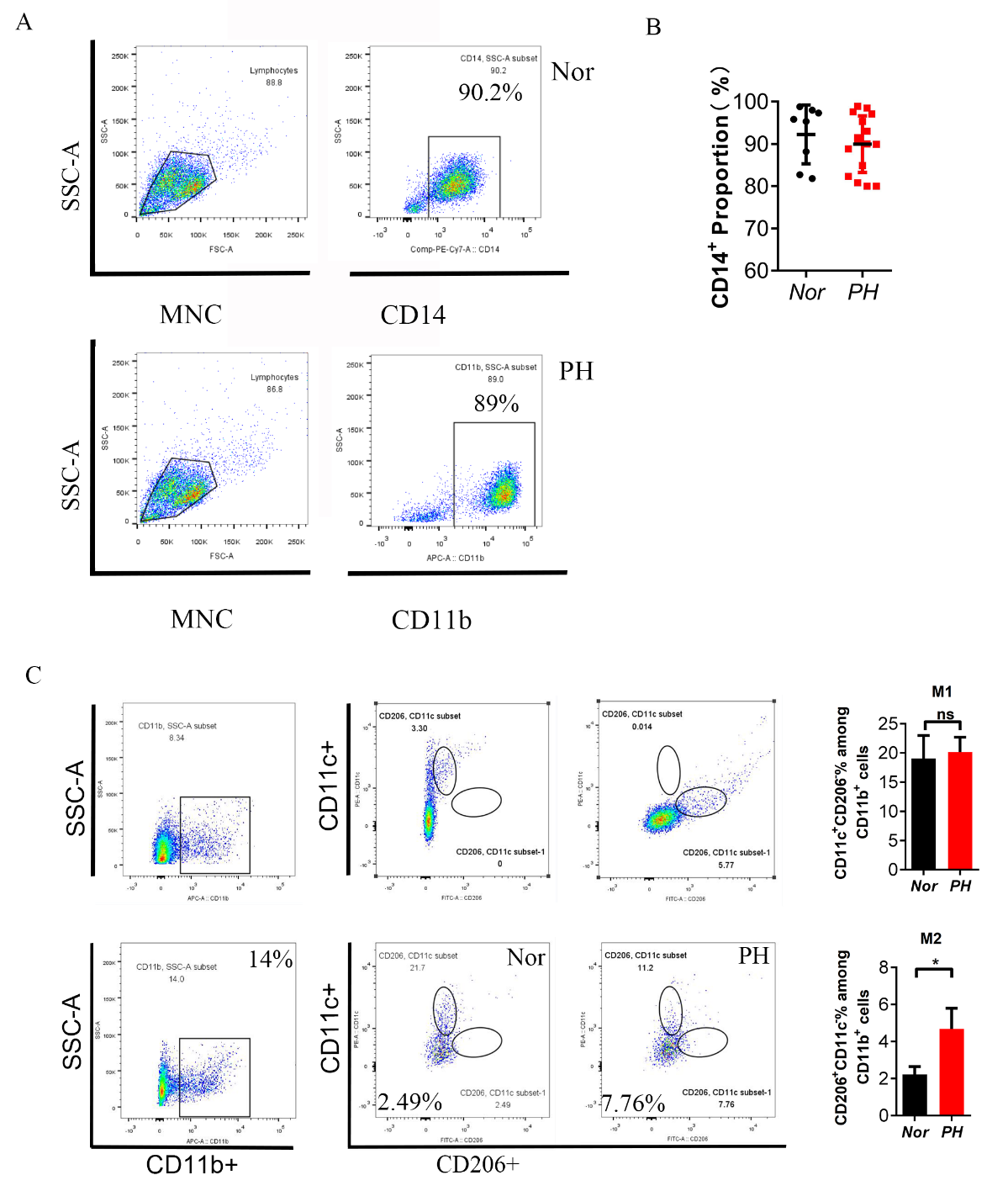


SFig.1 A: Spleen macrophages of patients were sorted by CD14^+^ magnetic beads， labeled with CD14^+^ and CD11b^+^ antibodies respectively, positive cells with similar purity . B: The purity of splenic macrophages sorted by CD14 magnetic beads in this study was greater than 80%.

The ratio of CD11c+ M1-like macrophages (CD11b^+^ CD11c^+^ CD206^-^) and CD206^+^ M2-like macrophages (CD11b^+^ CD206^+^ CD11c^-^) in splenocyte suspension was analyzed by FACS technique, and the significant difference between the two groups was counted (6 samples /group);

1. **Supplementary Figure 2**


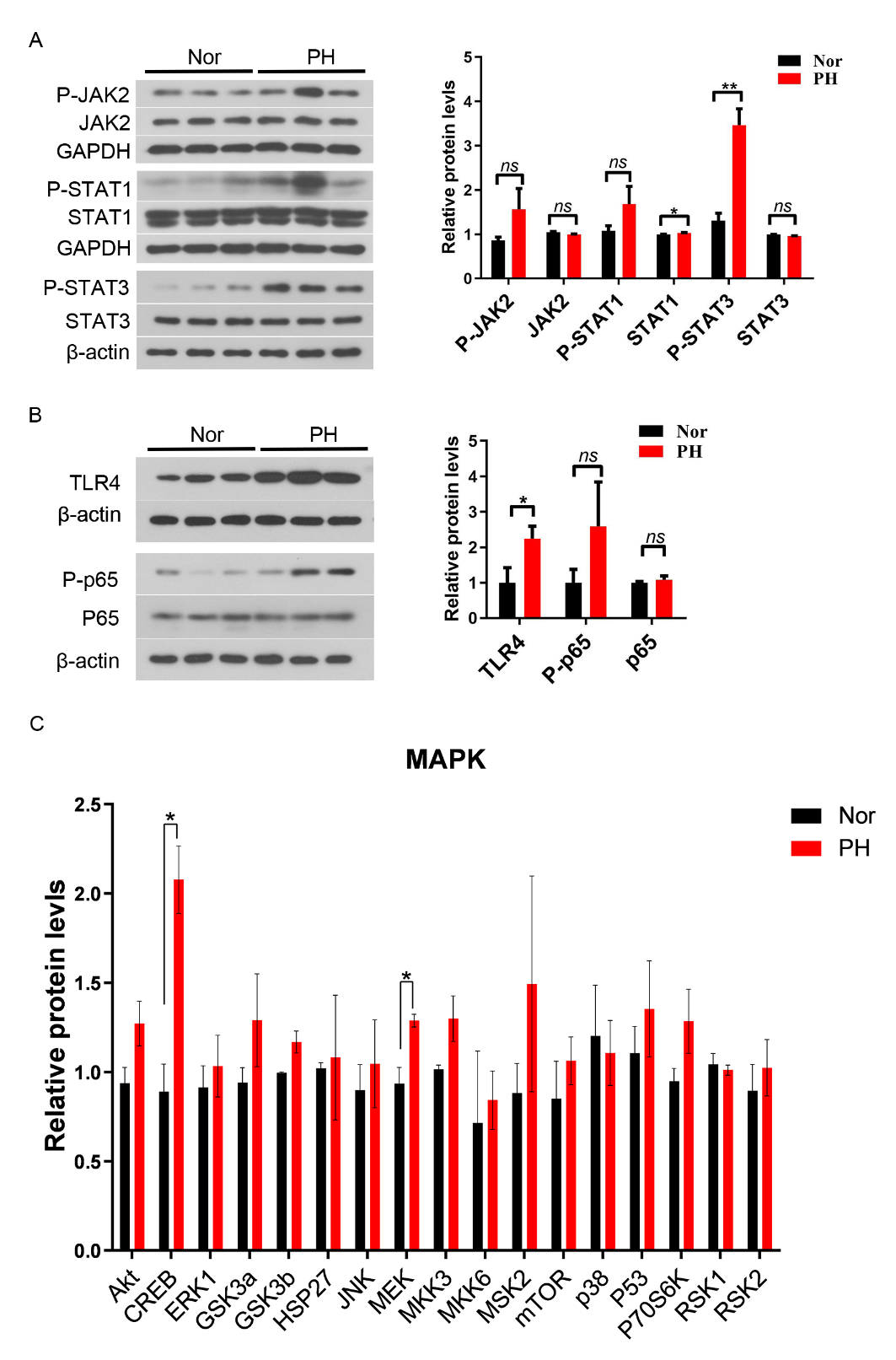


SFig.2：A-B. Western blot analysis the activation of JAK-STAT and TLR4/NF-κB signaling pathway in splenic macrophages of the Nor and PH groups (3 samples in each group). C. MAPK protein chip analysis of the expression of all detected proteins in splenic macrophages of the Nor and PH groups (2 samples in each group).

1. **Supplementary Figure 3**


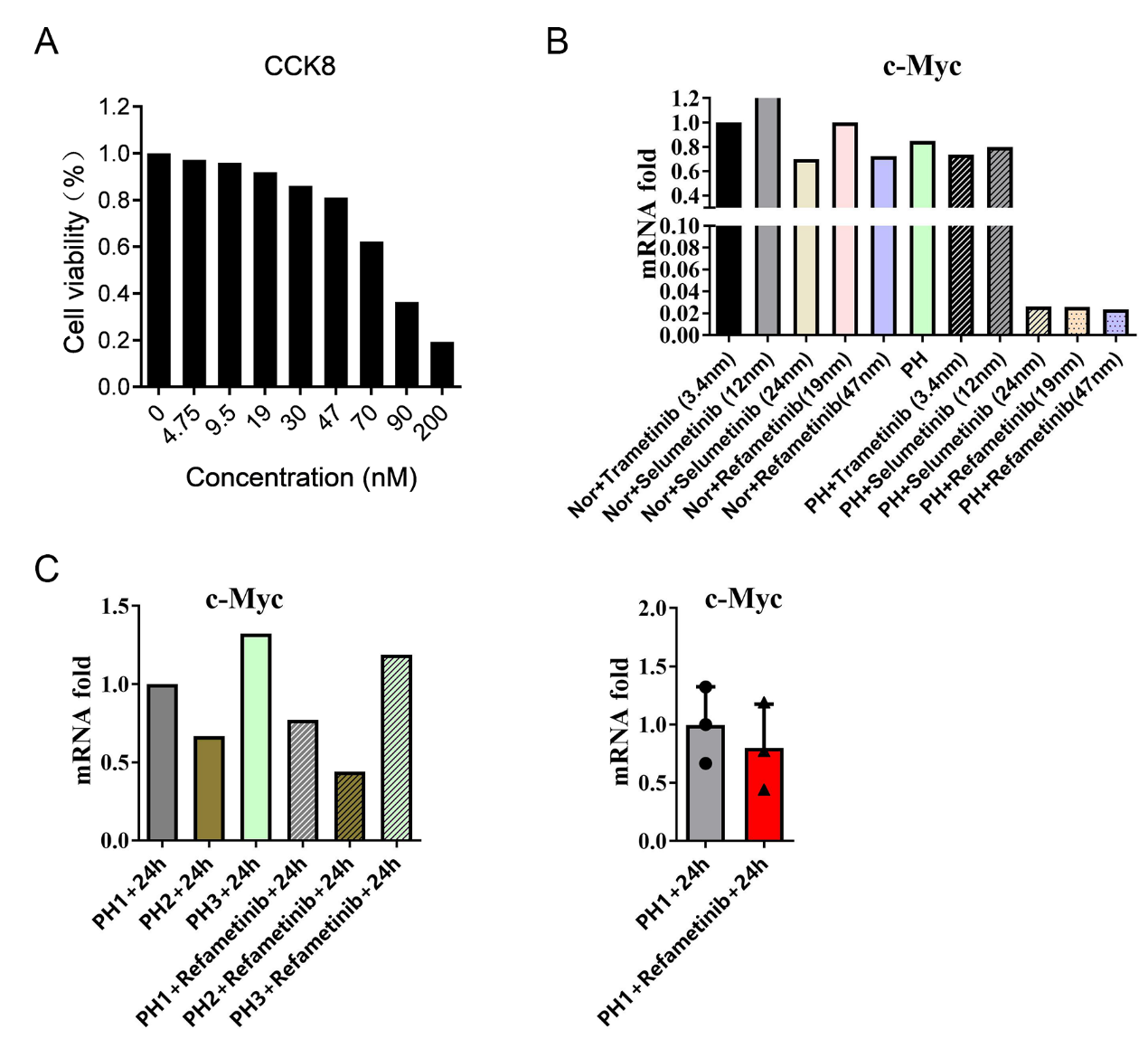


SFig.3: A. titrate the drug concentration of remifentanil (MEK inhibitor) with CCK8; B.Different MEK inhibitors were added to the mononuclear cells of Nor VS PH, and the expression of c-Myc was analyzed by qPCR; C. After adding Refametinib to different pH mononuclear cells, qPCR was used to analyze the downstream c-Myc expression.

1. **Supplementary Figure 4**


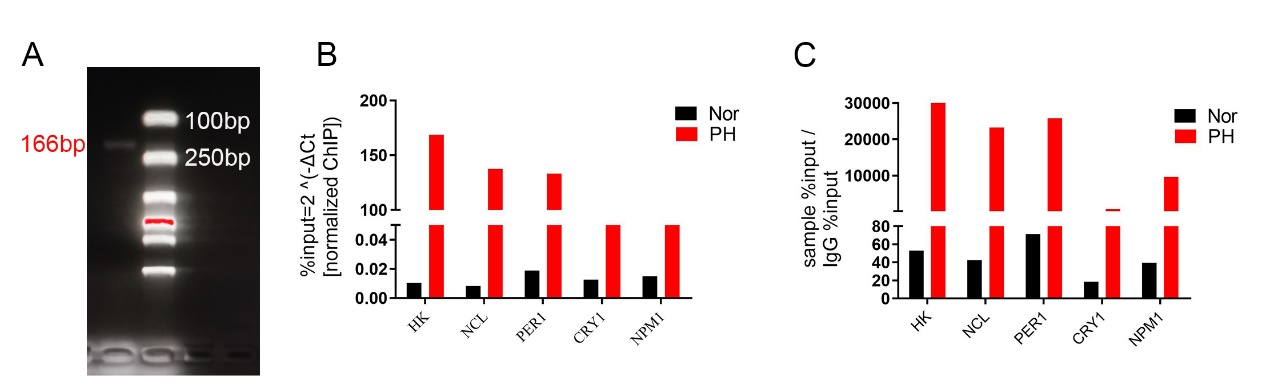


SFig.4: A. Perform electrophoresis analysis on the chip pull-down sample; B, C. Verify the expression of downstream binding genes related to c-Myc by qPCR.

1. **Supplementary Figure 5**


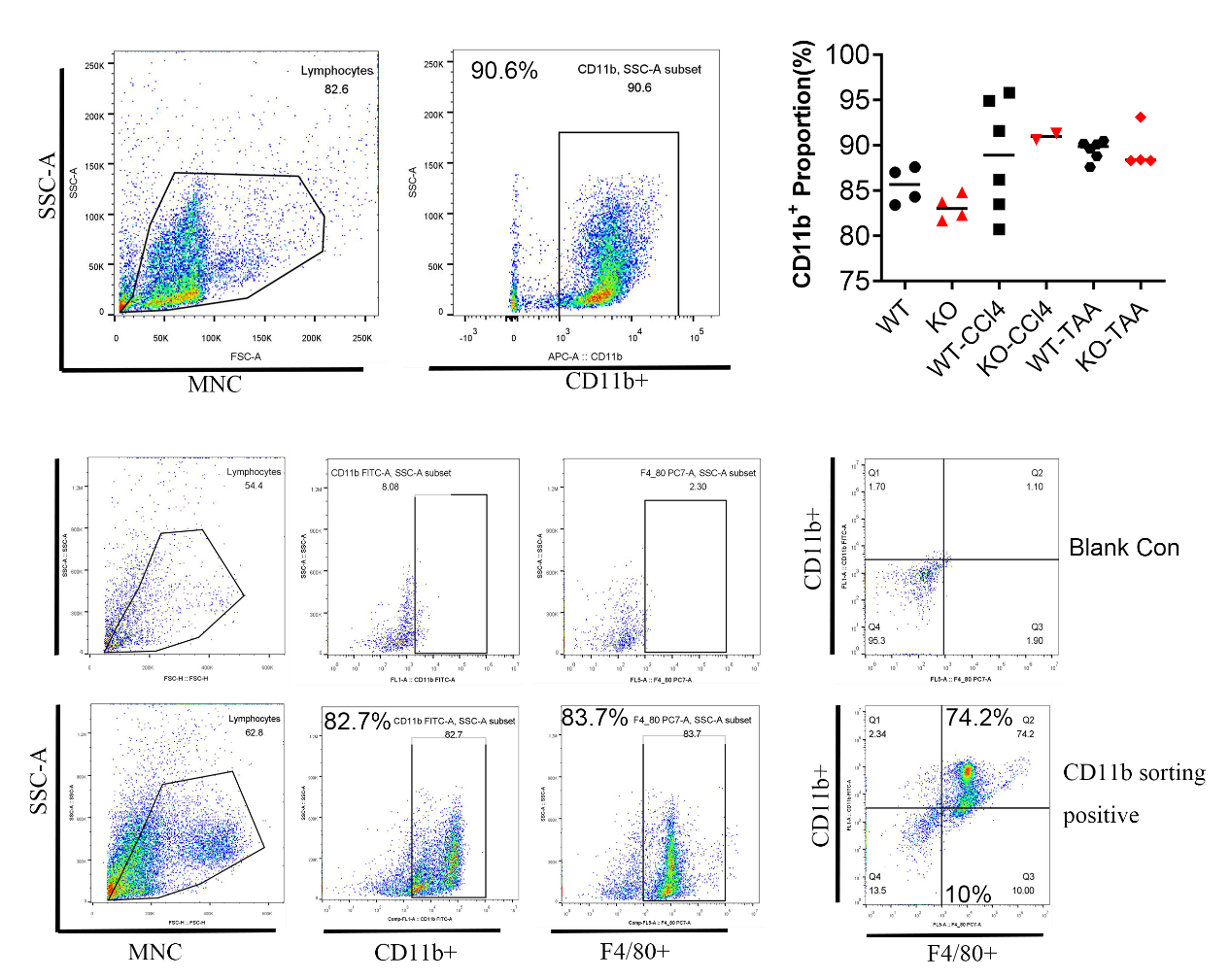


SFig.5: A-B. Use CD11b^+^magnetic beads to sort mouse spleen Monocyte-macrophages, label them with CD11b^+^and F4/80^+^antibodies, and calculate the proportion of CD11b^+^positive cells and F4/80^+^macrophages.

1. **Supplementary Figure 6**


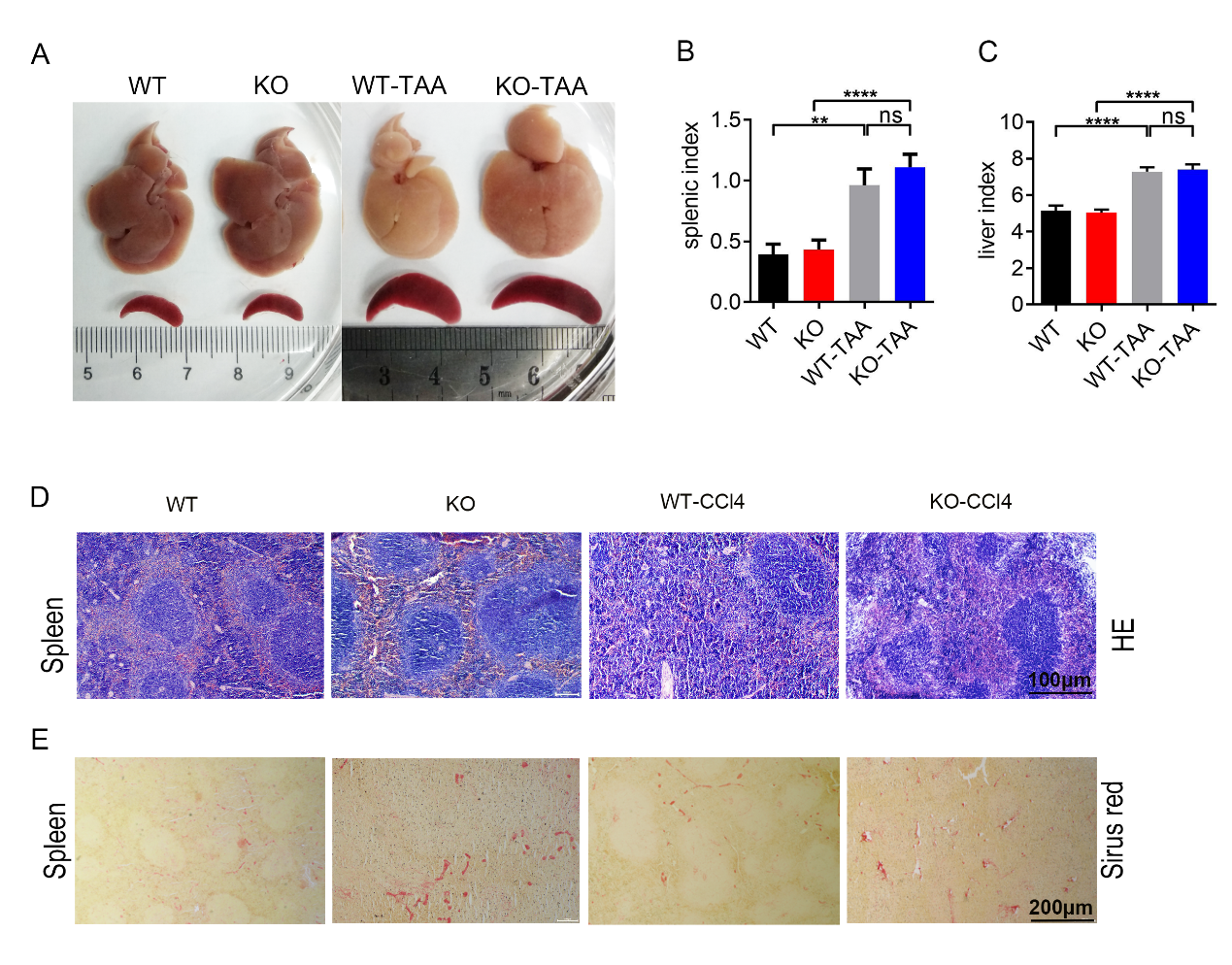


SFig.6: A-C. Changes of spleen index and liver index in TAA induced liver fibrosis model. D-E. HE staining and Sirius red staining of spleen tissue in CCL4 model mice。

1. **Supplementary Figure 7**


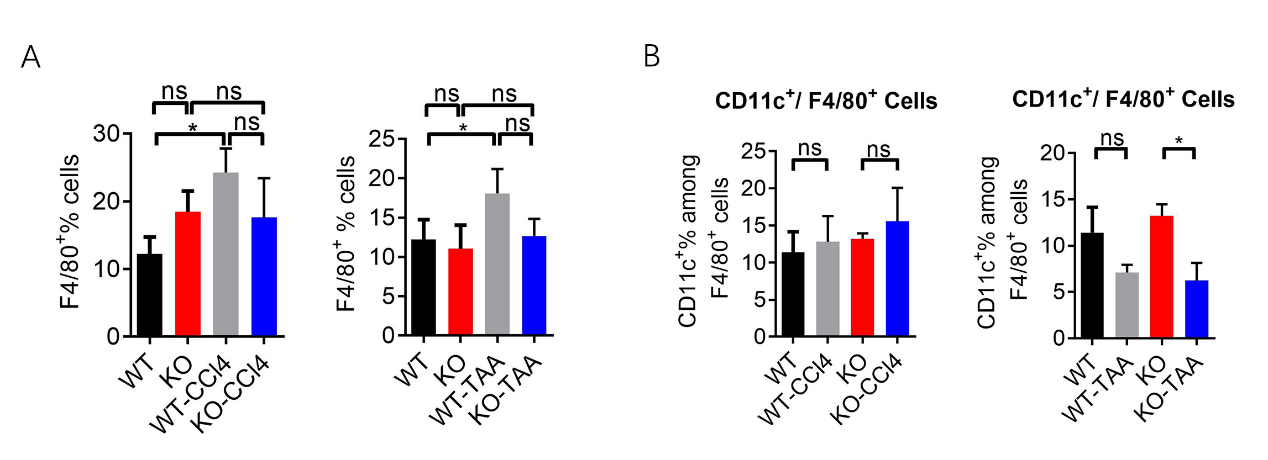


SFig.7: A, FASC analysis of changes in total macrophages (F4/80^+^) in each group after c-Myc knockout mouse CCl4 and TAA models; B, Changes in the proportion of M1 macrophages (F4/80^+^CD11c^+^CD206^-^).
